# Excretion of Urinary Orosomucoid 1 Protein Is Elevated in Patients with Chronic Heart Failure

**DOI:** 10.1371/journal.pone.0107550

**Published:** 2014-09-12

**Authors:** Li-na Hou, Fei Li, Qing-chun Zeng, Liang Su, Ping-an Chen, Zhi-hao Xu, Din-ji Zhu, Chang-hua Liu, Ding-li Xu

**Affiliations:** 1 State Key Laboratory of Organ Failure Research, Department of Cardiology, Nanfang Hospital, Southern Medical University, Guangzhou, Guangdong, P.R.China; 2 Department of healthy management, Nanfang Hospital, Southern Medical University, Guangzhou, Guangdong, P.R.China; 3 Department of Urology, Nanfang Hospital, Southern Medical University, Guangzhou, Guangdong, P.R.China; University of Udine, Italy

## Abstract

Easily screening markers for early detection of chronic heart failure (CHF) are lacking. We identified twenty differently expressed proteins including orosomucoid 1(ORM1) in urine between patients with CHF and normal controls by proteomic methods. Bioinformatics analyses suggested ORM1 could be used for further analysis. After verification by western blotting, the urinary levels of ORM1 were quantified with enzyme-linked immunosorbent assay (ELISA) by correcting for creatinine expression. The ORM1-Cr was significantly elevated in CHF patients than normal controls (6498.83±4300.21 versus 2102.26±1069.24 ng/mg). Furthermore, a Spearman analysis indicated that the urinary ORM1 levels had a high positive correlation with the classification of CHF, and the multivariate analysis suggested that the urinary ORM1 content was associated with the plasma amino-terminal pro- brain natriuretic peptide (NT-proBNP) (OR: 2.106, 95% CI: 1.213–3.524, *P = *0.002) and the New York Heart Association (NYHA) classification (OR: 3.019, 95% CI: 1.329–4.721, *P*<0.001). In addition, receiving operating curve (ROC) analyses suggested that an optimum cut-off value of 2484.98 ng/mg with 90.91% sensitivity and 85.48% specificity, respectively, could be used for the diagnosis of CHF. To sum up, our findings indicate that ORM1 could be a potential novel urinary biomarker for the early detection of CHF.

## Introduction

Chronic heart failure (CHF), a clinical syndrome of left ventricular systolic and/or diastolic dysfunction, remains a major threat to public health [1]. It has been estimated that there are currently over 5 million CHF patients, and more than 550,000 new patients diagnosed yearly in the USA [2]. With the ageing of the global population, the CHF populations are increasing. Recent studies have indicated that approximately 20% of people in the world will suffer CHF at some point through their lifetime [3]. Many CHF patients do not receive accurate diagnoses or optimum pharmacological treatments, therefore, resulting in substantial mortality and morbidity.

Although great progress has been made in diagnostic intensity and treatment, the long-term prognosis for CHF is still poor. In addition, the rising incidence of CHF is a cause for concern, and there is currently no gold standard for its diagnosis [4]. Early diagnosis and treatment are extremely important in this disease. Therefore, the development of a reliable, non-invasive biomarker is of considerable clinical importance, aiming at increasing the early detection rate of heart failure and/or predicting the progression of the disease in time.

Proteomic approaches, which could simultaneously analyze thousands of proteins and peptides, can be used to search for novel biomarkers for the early detection of the disease, their development and prognostic assessment, and the selection and monitoring of proper treatment modalities [5]. Proteomic patterns in body fluids present new opportunities for identification of novel, highly-sensitive specific markers for early detection of disease [6]. Obviously, urine samples are derived from minimally invasive procedures in clinical analysis, and recent advances in urinary proteomics have generated interest into new potential biomarkers for various systemic diseases such as various cancers and cardiovascular diseases [7], [8]. However, to date, easily applicable screening biomarkers for the early detection of CHF based on urine samples are lacking.

In our study, we applied two-dimensional differential gel electrophoresis (2D-DIGE) proteomic technologies to identify differentially expressed proteins in urinary protein extracts of CHF patients and healthy donors. Furthermore, we suggest that orosomucoid 1 (ORM1) is a potential novel urinary biomarker for the early detection of CHF.

## Material and Methods

### a) Urinary samples collection and preparation

All subjects in this study were recruited from the Chinese Han population at Nanfang Hospital (Guangzhou, China) from May 2011 to August 2012. The study protocol was approved by the Nanfang Hospital Ethics Committee. All subjects were informed about the purpose of the study and gave their written consent. In all, 197 patients with CHF were confirmed according to the Framingham Heart Study (FHS) diagnostic criteria of CHF [9]. The diagnosis of CHF was based on a complete clinical evaluation, laboratory testing, echocardiography, chest X-ray, and coronary angiography, coupled with plasma amino-terminal pro-brain natriuretic peptide (NT-proBNP) content ≥1000 pg/ml. The diagnosis was conducted independently by two experienced clinicians in Nanfang Hospital, Southern Medical University. Cardiac function for each CHF patient was divided into class II, class III, and class IV according to the Cardiac Function Standard of the New York Heart Association (NYHA). The main exclusion criteria were as follows: unconscious patients; other systemic diseases such as cancer, lung disease, liver cirrhosis, bleeding disorders, severe acute infectious and metabolic disorders. 83 healthy volunteers with no evidence of disease were used as control.

The second voided clean-catch urine samples form subjects were collected in the early morning. Each urine sample (20 ml) was directly collected into a sterile plastic tube and then immediately centrifuged at 2500×g for 10 min at 4°C to remove cell debris and particulate matter. The supernatant was stored at −80°C for further analysis. Repeated freeze-thaw cycles were avoided. All the clinical data of those subjects were collected as well.

### b) Proteomic analysis

Equal volume urine specimens from 15 CHF patients and 15 controls were pooled respectively for 2D-DIGE analysis. Of these CHF patients, there were five cases in each NYHA class (II, III and IV). For processing, samples were first thawed on ice with the addition of protease inhibitors, 1 mmol/L phenylmethylsulfonyl fluoride, 5 mmol/L phenanthroline, and 5 mmol/L benzamidine (Sigma, St. Louis, Missouri, USA), and then centrifuged using Centricon Plus-20, 10,000 MWCO devices (Millipore, Bedford, MA, USA). Following extraction, other interfering components in the concentrated urine were removed using a 2D Clean-Up Kit (GE Healthcare, Piscataway, New Jersey, USA) according to the manufacturer's instructions. Protein concentration was measured with the 2D Quant Kit (GE Healtcare, Uppsala, Sweden).

Protein extracts obtained from the pooled urine samples were labeled with Cy2, Cy3 and Cy5 dyes (CyDye DIGE Fluor minimal dyes, GE Healthcare) according to the Ettan two-dimensional difference gel electrophoresis (DIGE) protocol. Briefly, 50 µg of urine protein samples from CHF patients and control group were minimally labeled with 400 pmol of Cy5 and Cy3 fluorescent dyes respectively. An internal pool was labeled with Cy2 and used to assess the reproducibility and statistical inferences. It was generated by combining equal amounts of extracts from all CHF and healthy control samples included in the study. All the labeled reactions were incubated for 30 min on ice protected from light, and then stopped by adding 1 ml of 10 mM lysine for 10 min [10]. Following the labeling reaction, all the three labeled samples were mixed and resolved in one gel.

An immobilized pH gradient (IPG) strip (24 cm, pH 3–10, nonlinear, GE Healthcare) was used for isoelectric focusing. For rehydration, samples were brought to 450 µl with rehydration buffer and 1 ml of mineral oil was added to avoid evaporation. The IPG strips were passively rehydrated overnight at room temperature. Isoelectric focusing was performed according to the protocol provided by the manufacturer (GE Healthcare) as follows: 1 h at 500 V, 1 h at 1000 V, 1 h at 5000 V, 1 h at 8000 V, then maintaining at 8000 V until a total of 60 kVh. After focusing, the strips were prepared for the second dimension gels by incubation for 15 min in equilibrium buffers I and II, respectively. Standard continuous SDS-PAGE electrophoresis for the second dimension (12%) was carried out using an Ettan DALT twelve system (GE Healthcare) until the dye front reached the bottom of the gels. Following SDS-PAGE, image scans were performed immediately using a Typhoon 9410 scanner (GE Healthcare) at the excitation emission of 488/520 nm (Cy2), 532/580 nm (Cy3) and 633/670 nm (Cy5), respectively. In addition, another strip with 1000 mg of proteins loaded was performed in parallel as a preparative gel for spots picking as marked in 2D-DIGE. The gel was stained with Coomassie Brilliant Blue [11]. Identical samples were run in three times.

After scanning, the gel images were analyzed using the DeCyder 5.01 software (GE Healthcare). Its differential in-gel analysis (DIA) module was used for pairwise comparisons of each sample with the internal standard within each gel by calculating the normalized spot volumes. The spot volumes were calculated by the background intensities combined with the borders of the spots. Spot volumes were normalized by dividing each Cy3 or Cy5 spot volume with the corresponding internal standard (Cy2). The average abundance changes for each spot across the different spot maps were calculated with the DeCyder biological variation analysis (BVA) module. The spots whose ratios of Cy5/Cy2 and Cy3/Cy2 changed by 1.5-fold or greater were regarded as differently up- or down- regulated expressed spots and were considered for further protein identification.

As described in our previous studies [12], [13], the protein spots were identified by matrix-assisted laser desorption time-of-flight mass spectrometry (MALDI-TOF/TOF-MS). Shortly, the differentially expressed protein spots were excised from the Coomassie-stained gels with an Ettan Spot Picker (GE Healthcare), and then subjected to trypsin digestion, peptide extraction and desalting. The peptide mixtures were analyzed using an ABI Voyager DE-STR mass spectrometer (ABI 4700 Proteomic Analyzer, Applied Biosystems, Foster City, CA, USA). A trypsin- fragment peak was served as an internal standard for mass calibration. A list of the corrected mass peaks was the peptide mass fingerprinting (PMF).

Protein identification using peptide mass fingerprinting was performed using the MASCOT search engine (http://www.matrixscience.com/, Matrix Science Ltd, London, UK) against the MSDB protein database. The search was defined as the Homo sapiens subsets of the sequences in the Swiss–Prot and NCBI nonredundant protein sequence databases. The following search parameters were used during the searche: tolerance of one missed trypsin cleavages, the errors in peptide mass were within 25 ppm for both the precursor mass tolerance and the fragments mass. Carbamidomethylation of cysteine and oxidation of methionine as fixed and variable modifications, respectively, were taken into account for database searching. The proteins matched more than four peptides and with a MASCOT score higher than 63 were considered statistically significant (*P*<0.05). The identification results were filtered by peakErazor software (Lighthouse Data, Odense, Denmark). All matched sequences were manually validated [12], [13].

### c) Bioinformatic analysis

The interaction network of differentially expressed proteins was performed automatically by STRING (Search Tool for the Retrieval of Interacting Genes/Proteins; version 8.3; http://string.embl.de/) with following analysis parameters[14]: species — Homo sapiens, confidence level — 0.400, active prediction methods — all. The PubGene Analysis tool (http://www.pubgene.org/) was used to search for literature bio-association analysis of these proteins [15]. The bio-associations had the following categories in Pubgene: Process, Function and Component. The key bio-processes related to the identified proteins were retrieved for further analysis.

### d) Western blotting analysis

For western blot analyses, the samples involved six CHF patients (n = 2 in each class II, III and IV of NYHA), and six healthy controls. A total of 30 µg prepared urine proteins were separated by 12% SDS-PAGE. The gels were then transferred onto Polyvinylidene Xuoride (PVDF) (Millipore) membranes. The membranes were blocked in a solution of TBS containing 5% nonfat milk powder and 0.1% Tween-20 for 1 h at room temperature and then incubated overnight at 4°C with the monoclonal antibody against the human ORM1 protein (diluted 1∶500; Abcam, UK). After three 10-min washes in TBS-T, the membranes were incubated with horseradish peroxidase horseradish (HRP) conjugate of goat anti-rabbit IgG (Bioworld Technology, Louis Park, MN, USA) at a 1∶5000 dilution at room temperature for 1 h. The proteins were detected using an enhanced chemiluminescence (ECL, Pierce, Rockford, IL, USA) detection system. Relative intensities were documented and analyzed by densitometry.

### e) Enzyme-linked immunosorbent assay (ELISA) analysis

The concentration of ORM1 protein in urine samples was measured with a commercially available ELISA kit (R&D Systems, Minneapolis, USA) according to the manufacturer's instructions. The assay has a minimum detectable dose of ORM1 at 0.538 ng/ml, exhibiting linearity between 3.12 and 200 ng/ml. The standard curve was created using the suppliers' lyophilized human ORM1. The levels of urinary ORM1 were corrected by urinary creatinine (Cr) concentrations to avoid the influence of urine volume. Thereby, the results were expressed as ORM1 -to-Cr ratio (ORM1/Cr nanograms per miligrams of creatinine). Urine creatinine (Cr) levels were measured at the Department of Clinical Laboratory of Nanfang Hospital (Guangzhou, China).

### f) Statistical methods

All data were collected and used for statistical analyses with SPSS software 13.0 (SPSS, Chicago, IL, USA). Values were presented as mean ± standard deviation (SD). The difference among two groups or three groups was compared with Student's t test or one-way ANOVA. A Spearman analysis was performed to explore the relationship between urinary ORM1-Cr and the clinical characteristics of subjects where appropriate. Univariate (nonparametric rank sum test) and multivariate (logistic regression) analyses were conducted to evaluate the relationship between urinary ORM1 expression and clinical parameters of CHF, including age, sex, diabetes, hypertension, coronary heart disease, cardiomyopathy, renal dysfunction, left ventricular ejection fraction (LVEF), NT-proBNP and NYHA classification. Receiving operating curve (ROC) analyses were used to define the most optimal diagnostic cutoff as well as the diagnostic performance given by the area under the curve (AUC), estimating the sensitivity versus its false-positive rate at optimal cutoffs. The best statistical cut-off value of ORM1-Cr was defined, which means the point at which the sum of sensitivity and specificity is more than other points. The results were considered statistically significant at *P*-value <0.05.

## Results

### a) Clinical characteristics of subjects

Table 1 has list the clinical characteristics of all subjects in our study. There was no statistical difference in clinical characteristics (e.g. age, sex, serum creatinine and urinary creatinine). The urine samples form 15 CHF patients and 15 controls were randomly selected for 2D-DIGE analysis. Among the 15 CHF patients, there were five cases in each class II, III and IV of NYHA. Furthermore, there were 176 CHF patients and 62 healthy volunteers recruited for ELISA quantitative analysis. Of these 176 subjects with CHF, the CHF cases belonging to class II, III and IV numbered 34, 87 and 55 respectively.

**Table 1:** Clinical data for subjects in the study.

	Samples for 2D-DIGE		Samples for ELISA	
	Controls	CHF	Controls	CHF
Number	15	15	62	176
Gender (Male/Female)	8/7	6/9	30/32	99/78
Age (years)	58.67±14.89	61.73±13.82	59.82±13.17	62.62±14.59
Urine creatinine (mg/dl)	97.91±46.03	113.29±63.08	97.91±46.03	113.29±63.08
Serum creatinine (umol/L)	99.72±46.57	115.47±68.94	100.63±49.29	118.45±70.57
NT-proBNP (pg/ml)	/	6852.12±7816.69	/	7703.96±8695.85

### b) 2D-DIGE analysis and twenty differently expressed proteins were identified by MALDI-FOF/TOF-MS

**Figure 1:**
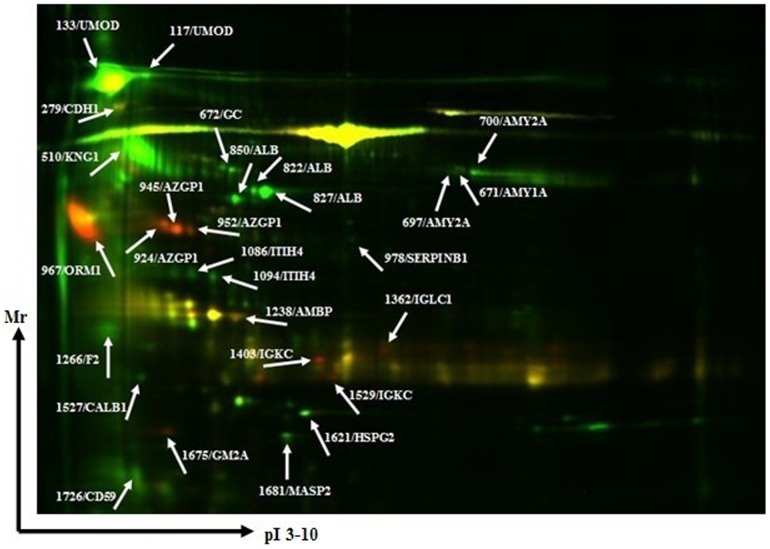
Distribution of all 28 differentially expressed protein spots in a representative two- dimensional fluorescent differential gel electrophoresis (2D-DIGE) image from proteomic profiling of urine samples from patients with CHF and healthy controls. Further identified protein spots are indicated by spot ID. Protein spots, protein name and spot ID are indicated (arrows).

Figure 1 has shown a representative 2D-DIGE image from proteomic profiling of urine samples from patients with CHF and healthy controls. After quantitative and statistical analysis, the 28 differential protein spots with volumes changed by 1.5-fold or more were selected for further identification by MALDI-FOF/TOF-MS. These 28 differently expressed protein spots corresponded to twenty different protein accession numbers (Fig. 1). Seven proteins were significantly up-regulated in CHF and the other thirteen were down- regulated. The up-regulated proteins were Cadherin-1 (CDH1), Zinc-alpha-2-glycoprotein (AZGP1), Alpha-1-acid glycoprotein 1 (ORM1), Protein AMBP (AMBP), Ig kappa chain C region (IGKC), Ig lambda-1 chain C regions (IGLC1),Ganglioside GM2 activator (GM2A). The down-regulated proteins involved Uromodulin (UMOD), Kininogen-1 (KNG1), Alpha-amylase 1 (AMY1A), Vitamin D-binding protein (GC), Pancreatic alpha-amylase (AMY2A), Serum albumin (ALB), Leukocyte elastase inhibitor (SERPINB1), Inter-alpha-trypsin inhibitor heavy chain H4 (ITIH4), Prothrombin (F2), Calbindin (CALB1), Basement membrane-specific heparan sulfate proteoglycan core protein (HSPG2), Mannan-binding lectin serine protease 2 (MASP2), CD59 glycoprotein (CD59). Information about these protein spots concerning access numbers, gene names, molecular weight, PI, total ion scores, fold changes of spots volumes and overall trends is presented in Table 2.

**Table 2:** Identification of differentially expressed proteins in urine from chronic heart failure and normal control.

Master number	Accession No.	Gene Name	Protein molecular weight	Protein PI	Pep. Count^a^	Total ion score	Fold changes^b^	Overall trend^c^
117	P07911	UMOD	72451.4	5.05	9	518	−12.77±3.46	down
133	P07911	UMOD	72451.4	5.05	9	323	−10.37±3.18	down
279	P12830	CDH1	97852	4.58	3	212	3.15±0.29	up
510	P01042	KNG1	72995.6	6.34	7	263	−2.54 ±0.33	down
671	P04745	AMY1A	58415.2	6.47	7	213	−3.05±2.19	down
672	P02774	GC	54525.6	5.4	3	134	−3.33±1.26	down
697	P04746	AMY2A	58354.3	6.6	8	106	−2.45±1.06	down
700	P04746	AMY2A	58354.3	6.6	8	215	−2.33±0.52	down
822	P02768	ALB	71317.2	5.92	8	208	−4.33±0.51	down
827	P02768	ALB	71317.2	5.92	9	229	−6.31±1.21	down
850	P02768	ALB	71317.2	5.92	7	163	−6.24±0.89	down
924	P25311	AZGP1	34079	5.57	5	265	7.31±1.54	up
945	P25311	AZGP1	34079	5.57	4	245	7.55±1.89	up
952	P25311	AZGP1	34079	5.57	6	349	7.46±2.27	up
967	P02763	ORM1	23724.8	4.93	3	302	14.81±3.49	up
978	P30740	SERPINB1	42828.7	5.9	5	233	−3.96±1.12	down
1086	Q14624	ITIH4	103521.1	6.51	4	189	−3.96±1.23	down
1094	Q14624	ITIH4	103521.1	6.51	5	212	−2.89±0.63	down
1238	P02760	AMBP	39886.3	5.95	4	298	5.63±2.03	up
1266	P00734	F2	71474.7	5.64	3	167	−4.55±1.96	down
1362	P01842	IGLC1	11400.6	6.92	1	32	−11.72±3.69	up
1403	P01834	IGKC	11772.7	5.58	1	94	17.63±5.67	up
1527	P05937	CALB1	30291.1	4.7	3	77	−4.09±0.86	down
1529	P01834	IGKC	11772.7	5.58	1	50	4.43±1.49	up
1621	P98160	HSPG2	479220.5	6.06	6	253	−2.89±1.34	down
1675	P17900	GM2A	21280.9	5.17	2	152	6.31±3.63	up
1681	O00187	MASP2	77224.2	5.47	7	351	−3.15±1.74	down
1726	P13987	CD59	14795	6.02	1	59	2.99±1.36	down

a Calculated by amino acid count.

b Fold changes of spot intensities represented as mean ± SD.

c up: up-regulated in the heart failure group. down: down-regulated in the heart failure group.

### c) ORM1 was indicated for further investigation based on bioinformatics analysis

**Figure 2:**
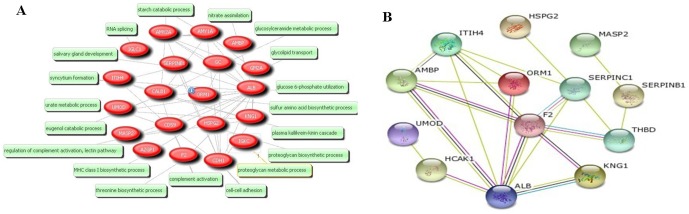
Bioinformatic analysis of bio-association network by Pubgene and String programs. (A) Bio-process associated with the twenty identified proteins was indicated by Pubgene. (B)The ORM1 protein function partner network was indicated by String analysis.

Pubgene analysis of the twenty identified proteins was performed to search for the associated bio-processes. As a result, this analysis predicted 1,600 different bio-processes to be associated with identified proteins. The top 15 closely related bio-processes included growth, induction, signal transduction, pathogenesis, secretion, catabolic process, digestion, localization, translation, coagulation, death, RNA splicing, apoptosis, phosphorylation and complement activation. Noticeably, ORM1 was predicted to play important roles in many of these 15 bio-processes such as growth, induction, signal transduction, pathogenesis, catabolic process, coagulation and phosphorylation (Table 3). Furthermore, we observed tight connections between ORM1 and other identified proteins such as ALB, AMBP and ITIH4 from the Network based on co-occurrence in articles, which was consistent with the result by STRING 9.0 program. Those findings suggested that ORM1 was a key function partner with ALB, AMBP and ITIH4 (Fig. 2B), which play vital roles in the development of acute-phase reaction and inflammation indicated by Pubgene (not shown).

**Table 3:** Top 15 of total 1600 bio-process for the twenty identified proteins in Pubgene.

Description	Associated Terms	Article (*p*-value)
Growth	ALB, AMBP, AMY1A, AMY2A, AZGP1, CALB1, CD59, CDH1, F2, GC, GM2A, HSPG2, IGKC ITIH4 KNG1, MASP2, ORM1, SERPINB1, UMOD	7.96
Induction	ALB, AMBP, AMY1A, AMY2A, AZGP1, CALB1, CD59, CDH1, F2, GC, GM2A, HSPG2, IGKC, ITIH4, KNG1, MASP2, ORM1, SERPINB1, UMOD	3.97
Signal transduction	ALB, AMBP, AMY1A, AMY2A, AZGP1, CALB1, CD59, CDH1, F2, GC, GM2A, HSPG2, IGKC, ITIH4, KNG1, MASP2, ORM1, SERPINB1, UMOD	3.93
Pathogenesis	ALB, AMBP, AMY1A, AMY2A, AZGP1, CALB1, CD59, CDH1, F2, GC, GM2A, HSPG2, IGKC, ITIH4, KNG1, MASP2, ORM1, SERPINB1, UMOD	3.76
Secretion	ALB, AMBP, AMY1A, AMY2A, CALB1, CD59, CDH1, F2, GC, GM2A, HSPG2, ITIH4, KNG1, SERPINB1, UMOD	3.15
Catabolic process	ALB, AMY1A, AMY2A, AZGP1, CALB1, CD59, CDH1, F2, GC, GM2A, HSPG2, IGKC, ITIH4, KNG1, ORM1, SERPINB1, UMOD	3.1
Digestion	ALB, AMY1A, AMY2A, AZGP1, CALB1, CD59, CDH1, F2, GC, GM2A, HSPG2, IGKC, ITIH4, KNG1, MASP2, SERPINB1, UMOD	2.49
Localization	ALB, AMY1A, AMY2A, AZGP1, CALB1, CD59, CDH1, F2, GC, GM2A, HSPG2, IGKC, ITIH4, KNG1, MASP2, SERPINB1, UMOD	2.44
Translation	ALB, AMBP, AMY1A, AMY2A, AZGP1, CALB1, CD59, CDH1, F2, GC, GM2A, HSPG2, IGKC, ITIH4, KNG1, MASP2, SERPINB1, UMOD	1.95
Coagulation	ALB, AMY1A, AMY2A, CD59, CDH1, F2, GC, HSPG2, ITIH4, KNG1, MASP2, ORM1, SERPINB1, UMOD	1.53
Death	ALB, AMY1A, AMY2A, AZGP1, CALB1, CD59, CDH1, F2, GC, GM2A, HSPG2, IGKC, ITIH4, KNG1, MASP2, SERPINB1, UMOD	1.33
RNA splicing	ALB, CDH1, IGKC, IGLC1	1.1
Apoptosis	ALB, AMBP, AMY1A, AMY2A, CALB1, CD59, CDH1, F2, GC, HSPG2, ITIH4, KNG1, SERPINB1, UMOD	1.04
phosphorylation	ALB, AMY1A, AMY2A, CALB1, CD59, CDH1, F2, GC, HSPG2, IGKC, ITIH4, KNG1, ORM1, UMOD	1
complement activation	ALB, CD59, CDH1, ITIH4, KNG1, MASP2	0.982

*P* =  term/total (number of records of terms according to a specific function divided by their total number of records in the MEDLINE).

### d) Verification of higher urinary ORM1 levels in patients with CHF

**Figure 3:**
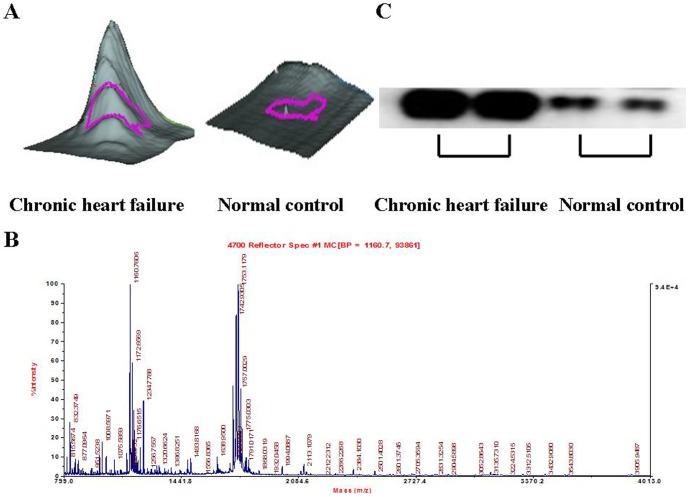
Verification of ORM1 protein expression in urine samples form patients with CHF and normal controls. (A) A 3-D view of ORM1 protein was to show the higher expression of ORM1 protein in urine of patients with CHF compared with normal controls. (B) Verification of ORM1 expression in individual urine samples of patients with CHF and normal controls by western blotting. (C) MS of in-gel trypsin digests of the protein and analysis of the depicted peptide spectrum resulted in the identification of ORM1.

To further verify our proteomic findings, we used western blotting analysis to study the expression of urinary ORM1 in individual samples from patients with CHF and control groups. The results clearly demonstrated that urinary ORM1 was significantly up-regulated in CHF cases in comparison to controls. These variations in urinary ORM1 expression were consistent with our DIGE results (Fig. 3).

### e) Quantification of urinary ORM1 by ELISA

**Figure 4:**
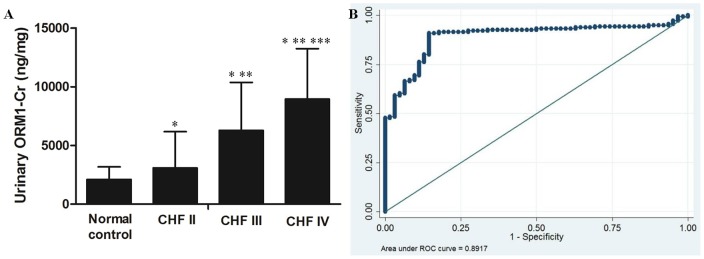
Urinary ORM1 as a potential biomarker for CHF. (A) ELISA was used to quantify urinary ORM1 levels in 176 patients with CHF and 62 healthy controls. The expression level of ORM1 in different NYHA functional classification of CHF. *P <0.05 compared with control group; **P <0.05 compared with CHF I; ***P <0.05 compared with CHF III. (B) ROC curve of urinary ORM1 as a detection marker for CHF was based on a series of 238 urine samples. The optimal cut off was 2484.98 ng/mg, and the area under the ROC curve (AUC) for diagnosis of CHF was 0.892 (95% confidence interval (CI) 0.848–0.936).

ELISA was used to quantify urinary ORM1 levels in 176 patients with CHF and 62 healthy controls. After normalization by creatinine level, the urinary ORM1 was markedly elevated in patients with CHF compared to controls (6498.83±4300.21 versus 2102.26±1069.24 ng/mg, *P*<0.0001) (Fig. 4A). When the CHF patients were stratified by NYHA classification, the urinary ORM1 concentrations were 3086.24±1474.91, 6284.97±4088.02 and 8946.71±4298.05 ng/mg for CHF patients with class II, III and IV, respectively. The expression level of urinary ORM1 was positively associated with the class of CHF classification (*P*<0.0001). The correlation indicated by the Spearman test is 0.499, suggesting that the level of ORM1 was markedly elevated in urine as heart failure worsened. In addition, the relationship between urinary ORM1 levels and the clinical characteristics of patients with CHF is presented in Table 4. There was no significant association between the urinary ORM1 expression and other clinical features of CHF such as age, sex, diabetes, hypertension, coronary heart disease, cardiomyopathy, renal dysfunction and left ventricular ejection fraction (LVEF) except for NT-proBNP and the classification of CHF. Furthermore, the multivariate analysis suggested that the urinary ORM1 content was associated with the NT-proBNP (OR: 2.106, 95% CI: 1.213–3.524, *P = *0.002) and the NYHA classification of CHF (OR: 3.019, 95% CI: 1.329–4.721, *P*<0.001) (Table 5).

**Table 4:** Relationship between urinary ORM1/Cr level and chronic heart failure.

Clinical features	Number	Urinary ORM1 levels (ng/mg)	*P*
age≥65 years	83	6608.92±4207.72	0.750
age<65 years	93	6400.57±4401.53	
Male	108	6254.27±4292.88	0.343
Female	68	6887.25±4314.96	
With diabetes	28	6284.09±4181.24	0.664
Without diabetes	148	6437.29±4333.47	
With hypertension	68	5894.90±3791.18	0.140
Without hypertension	108	6879.08±4567.93	
With coronary heart disease	62	6885.65±4416.60	0.380
Without coronary heart disease	114	6288.45±4240.41	
With cardiomyopathy	42	6033.04±3915.83	0.423
Without cardiomyopathy	134	6644.82±4417.51	
With renal dysfunction	31	7503.97±4472.75	0.152
Without renal dysfunction	145	6283.94±4247.46	
LVEF<50%	136	6922.04±4432.13	0.153
LVEF≥50%	40	5990.78±4106.26	
NT-proBNP≥7704* pg/ml	62	8344.42±4719.76	<0.001
NT-proBNP<7704 pg/ml	114	5495.09±3705.04	
NYHA II	34	3086.24±1474.91	<0.001
NYHA III	87	6284.97±4088.02	
NYHA IV	55	8946.71±4298.05	

* The mean value of NT-proBNP in CHF patients.

**Table 5:** The multivariate analysis of urinary ORM1/Cr level and clinical parameters.

Parameter	*p*-value(uni)	*p*-value(multi)	95% CI	OR
Age	0.815	0.637	0.563–1.426	1.012
Sex	0.567	0.346	0.852–1.639	1.156
Diabetes	0.342	0.266	0.899–1.963	1.818
Hypertension	0.459	0.501	0.603–2.507	1.962
Coronary heart disease	0.147	0.193	0.786–3.722	2.321
Cardiomyopathy	0.861	0.632	0.479–1.892	1.523
Renal dysfunction	0.201	0.153	0.463–1.557	0.678
LVEF	0.747	0.518	0.902–1.010	0.998
NT-proBNP	<0.001	0.002	1.213–3.524	2.106
NYHA classification	<0.001	<0.001	1.329–4.721	3.019

### f) Evaluation of urinary ORM1 as a potential biomarker for CHF

After quantitative measurement of ORM1 in 238 urine samples by ELISA, we applied ROC curves to assess the potential utility of urinary ORM1 in diagnosing and monitoring CHF. ROC analyses rendered an optimum cut-off value of 2484.98 ng/mg corresponding to 90.91% sensitivity and 85.48% specificity. The area under the ROC curve (AUC) of ORM1-Cr for diagnosis of CHF was 0.892 (95% confidence interval (CI) 0.848–0.936).

## Discussion

The exact incidence and prevalence of CHF remain probably underestimated due to the difficulties in accurate diagnosis [16]. Easily applicable screening markers for the early detection of the clinical course of CHF are currently lacking. A number of published studies have used proteomic methods to identify novel biomarkers for a variety of diseases. Notably, urinary proteomic biomarker models have recently been developed and shown potential for the accurate identification of several different disorders including ischaemic heart disease [17], endometriosis [18], diabetic nephropathy [19] and some cancers [20], [21].

In our present study, we focused on the urinary proteome, because urine is easy to collect relatively large quantities using non-invasive procedures compared with other body fluids. Moreover, the clinical importance of some urinary proteins has been demonstrated not only in urogenital diseases, but also in other systemic diseases [22]–[24]. Here, we conducted a 2D-DIGE analysis, which is an accurate quantitative comparison proteomic method, to compare the different protein expression profiles in the urine proteomes between CHF patients and healthy controls. Twenty differentially expressed proteins were identified as candidate biomarkers correlated with CHF. We further identified ORM1 as a novel CHF-related biomarker, which was significantly up-regulated in CHF patients, by bioinformatic analysis, western blotting and ELISA analyses.

Our bioinformatic analyses indicated that ORM1 played important roles in many bio-processes such as growth, induction, signal transduction, pathogenesis, catabolic process, coagulation and phosphorylation. ORM1 was also suggested to be a key function partner with ALB, AMBP and ITIH4 (Fig. 2B). Furthermore, inflammation was demonstrated to be associated with the pathophysiology of CHF [25]. ORM1 is a prominent component of the temporary proteinuria, which could be associated with exercise and acute inflammation [26]. To date, a study regarding the association between urinary ORM1 and CHF has not been reported. Thus, ORM1 was selected for further investigation in our study.

ORM1 (Orosomucoid-1, α1-acid glycoprotein 1), a single-chain polypeptide with a high carbohydrate moiety (42%) and a strong negative charge (isoelectric point, 2.7), functions as a transport protein in the bloodstream [27], [28]. It binds various ligands in the interior of its beta-barrel domain, and also binds synthetic drugs and influences their distribution and availability in the body. Human liver cells are normally the site of ORM1 production, but it can also be produced in endothelial cells and some tumor cells [29]. Some studies have demonstrated that ORM1 could be also synthesized by lymphocytes, macrophages, monocytes and granulocytes [26], [30]. ORM1, a member of immunocalins family, has a regulatory dampening effect on the inflammatory cascade, thereby protecting against tissue damage from excessive inflammation [28], [31]. Some studies have demonstrated that the concentrations of ORM1 in plasma and urine are usually much lower than albumin, but increased in urine to concentrations equal to or higher than albumin in conditions of acute inflammation and tissue repair [32]–[34].

To the best of our knowledge, this study is the first time that ORM1 has been identified in urine from patients with CHF by 2D-DIGE proteomic means. Our urinary proteome findings showed that the expression of ORM1 was significantly increased 14.81-fold in the CHF group compared with healthy controls. Meanwhile, the expression of elevated ORM1 in urine from CHF patients was further confirmed by western blotting. After quantitative ELISA analysis of urinary ORM1 in 238 individual samples, we observed that the levels of urinary ORM1 were elevated in CHF patients than controls, with significant difference found. Furthermore, the Spearman analysis showed that urinary ORM1 had a high positive correlation with the NYHA functional classification of CHF, indicating that the increased level of urinary ORM1 was associated with the CHF worsened, and the multivariate analysis indicated that the urinary ORM1 expression correlated with the plasma NT-proBNP (*P = *0.002) and NYHA classification (*P*<0.001). In addition, ROC curves suggested at an optimum cut-off value of 2484.98 ng/mg with 90.91% sensitivity and 85.48% specificity respectively could be used for the diagnosis of CHF. Thus, all those results demonstrate the urinary ORM1 protein has potential value for the early diagnosis of CHF.

However, the pathophysiologic mechanisms responsible for those findings are unclear. One possible explanation could be that the increased ORM1 may be correlated with the inflammatory activation in patients with CHF. Previous studies have provided solid evidence to support a pivotal role of inflammation in the underlying pathophysiology of CHF through both animal and human research [35], [36]. Cytokines are mainly produced by activated monocytes and macrophages, and these inflammatory proteins have formed the basis of the CHF inflammatory paradigm. Notably, monocyte activation, which plays a core role in the inflammatory pathophysiology of CHF, results in the subsequent release of inflammatory cytokines, their migration to the myocardium, adhesion to the endothelial wall, and other complex processes of the immune system [36], [37]. It has been demonstrated that C-reactive protein levels are increased in patients with CHF and could activate monocytes and then stimulate their production of inflammatory cytokines in a dose-dependent manner. Moreover, this cytokine production is significantly enhanced in those patients with ongoing myocardial damage than in those without [37], [38]. Thereby, we suppose that the levels of urinary ORM1, similar to the C-reactive protein, may be closely related to inflammatory condition of CHF. Further studies are needed to confirm the reasons leading to the exaggerated excretion of urinary ORM1 in CHF patients.

Most studies have investigated the levels of ORM in serum. Only recently have some studies begun to look at changes in ORM levels in the urine of patients with certain diseases. Christiansen *et al*. [39] reported that levels of ORM were found to be significantly elevated in the urine of patients with type 2 diabetes, even in normoalbuminuric patients. Furthermore, they suggested that the increased excretion of urinary ORM could be an independent, powerful predictor of cardiovascular mortality in patients with type 2 diabetes, as determined through five years of follow-up [30]. Their explanation was that the elevated urinary ORM is associated with cardiovascular risk factors such as inflammation, impaired left ventricular function and endothelial dysfunction [40]. Exaggerated excretion of urinary ORM was also found to predict preeclampsia in pregnant women with pregestational type 1 diabetes, which was probably caused by an increased local renal inflammatory response [41]. The enhanced urinary ORM concentrations were also detected in hypertensive subjects, along with other proteins such as albumin, a-1-antitrypsin, transferrin and retinol binding proteins [42]. In addition, Irmak S *et al*. [43] identified that ORM is significantly increased in urine samples from bladder cancer patients, particularly in those with invasive tumor stages.

Several limitations of the current study, especially about the specificity of this potential biomarker, are needed to be considered when contemplating the potential value of urinary ORM1 in early diagnosis of CHF. First, all the participants were recruited from Chinese Han populations in the same hospital. As a result, our sample must be regarded as a homogeneous cohort that may not represent the overall CHF population. Second, although we did not detect an association between urinary ORM1 levels and other clinical characteristics of CHF such as age, sex, diabetes, hypertension, coronary heart disease, cardiomyopathy, renal dysfunction and LVEF, we could not exclude the possibility that other unknown factors may affect the urinary ORM1 expression. Another point that needs to be addressed is the control group. Inflammation could lead to an elevated level of ORM, and healthy controls made up the only control group in our study, probably resulting in an overestimation of the specificity of ORM1. Therefore, our preliminary findings require further exploration through larger-scale studies with adequate control groups, in particular controls that include subjects with inflammation.

Above all, this study firstly reports the identification and validation of the significantly up-regulated protein of urinary ORM1 in CHF. The elevated urinary ORM1 levels were detected in patients with CHF than healthy controls. Moreover, urinary ORM1 expression had a high positive correlation with the NYHA functional classification of CHF. Thus, our findings suggest that urinary ORM1 could be a potential biomarker for detecting CHF. Further research is warranted to investigate the possible role of urinary ORM1 in the pathogenesis of CHF.
